# Association between weight-adjusted waist circumference index and depression in older patients with hypertension: a study based on NHANES 2007–2016

**DOI:** 10.3389/fpubh.2024.1461300

**Published:** 2024-09-13

**Authors:** Yi Niu, Yuqin Sun, Yijie Xie, Shun Yu

**Affiliations:** ^1^Wuxi Medical College of Jiangnan University, Wuxi, China; ^2^Department of Wound Stoma Care Clinic, Affiliated Hospital of Jiangnan University, Wuxi, China; ^3^Department of Plastic Surgery, Treatment Center of Burn and Trauma, Affiliated Hospital of Jiangnan University, Wuxi, China

**Keywords:** hypertension in the older adult, WWI index, depression, cross-sectional study, NHANES

## Abstract

**Objective:**

Our aim was to assess the relationship between weight-adjusted waist circumference index (WWI) and the prevalence of depression in older adult hypertensive patients in the United States.

**Methods:**

We selected individuals from the National Health and Nutrition Examination Survey (NHANES) database from 2007 to 2016 and used logistic regression analyses, subgroup analyses, and dose–response curves to assess the associations between the WWI index and the prevalence of depression in older hypertensive patients with age, sex, and BMI.

**Results:**

A total of 4,228 participants aged ≥60 years with hypertension were included in our study; 364 patients were assessed for depression. After correction for confounders, each unit increase in WWI increased the risk of depression in older hypertensive patients by 19% (OR = 1.19, 95% CI: 0.99, 1.43). Dose–response curves showed that the WWI index was positively associated with the prevalence of depression in older hypertensive patients when the WWI index was ≥11.6. Based on subgroup analyses, this association was particularly pronounced in individuals ≥70 years of age, women, and individuals with a BMI of 25 or greater.

**Conclusion:**

Higher WWI scores were positively associated with the prevalence of depression in older hypertensive patients and correlated with gender, age and BMI. This is notable, although a causal relationship cannot be established at this time.

## 1 Introduction

Hypertension is one of the highest global disease burdens and is widely recognised as a major risk factor for cardiovascular disease (1). According to the World Health Organization (WHO), approximately 1.13 billion people worldwide currently suffer from hypertension, and the number of people with hypertension is expected to increase by more than 400 million by 2025 (2). Lifestyle changes such as lack of exercise and obesity are the main reasons for developing high blood pressure (3). Hypertension in older adults is associated with adverse cardiovascular outcomes including heart failure, stroke, myocardial infarction and death. The global burden of hypertension is increasing due to population ageing and the rising prevalence of obesity, and is expected to affect one third of the world’s population by 2025 (4). The China Cardiovascular Health and Disease Report 2021 (5) also points out that the prevalence of hypertension in China continues to increase with socioeconomic development and the accelerated ageing of the population, especially in rural areas. There are currently 245 million hypertensive patients in China, and this number is increasing, placing a growing economic burden on the population and society. Although the rates of awareness, treatment and control of hypertension have increased, they remain low overall.

Depression is the most common mental illness in the world. It has become a major public health problem, with the number of cases of depression increasing globally from 172 million cases in 1990 to 25.8 million cases in 2017, an increase of 49.86% (6). Major depressive disorder (MDD) and generalised anxiety disorder (GAD) are leading causes of disability and premature death. Worldwide, it is estimated that more than 300 million people suffer from major depressive disorder, representing 4.4% of the world’s population (7). Depression, as a highly prevalent condition in the older population, not only seriously affects the quality of life of older people, but the presence of depression in older people is associated with a poor prognosis for many medical conditions, leading to a disproportionate burden of care, high rates of disability and mortality (8). Depression is a common disorder in patients with cardiovascular disease, with a prevalence of between 20 and 45%, much higher than in the general population (9). Studies have shown that the high incidence of depression not only affects the management and prognosis of chronic diseases such as hypertension, coronary heart disease and diabetes mellitus, but is also strongly associated with morbidity and mortality from cardiovascular disease (10).

High blood pressure (hypertension) is a common chronic condition in older people that can seriously affect their physical and mental health, causing anxiety, depression and other symptoms (11). In addition, the daily use of medication and the fear of serious complications can also be a source of psychological distress for patients (12). Depression prevalence among hypertensives in India 39.8% (13). The prevalence of depression among hypertensive patients in Ethiopia was 32.43% (14). Previous studies suggest that depression is common in patients with hypertension, which, if untreated, increases the risk of cardiovascular mortality and morbidity (15). When older adults have both hypertension and depression, the double burden of physical and mental illness increases the risk of cardiovascular disease, medication non-adherence, poor quality of life and suicide (16, 17). In addition, studies have shown that depression affects treatment, physical functioning and health outcomes in people with hypertension. People with chronic hypertension often have mood disorders, but few are able to recognise and properly understand the severity of depression in time, leading to delays in treatment, which can also affect hypertension control (18).

Depression and obesity are 2 common disorders that have been repeatedly shown to be associated with each other (19). The Weight Adjusted Waist Index (WWI) is an innovative measure of obesity that appears to outperform the Body Mass Index (BMI) and Waist Circumference (WC) in assessing lean and fat mass (20). The conventional wisdom is that obesity is detrimental to the health of older people. Obesity is associated with a number of diseases, including type 2 diabetes (21), hypertension (22), dyslipidemia (23) and ischaemia heart disease (24). These conditions not only affect the quality of life of older people, but can also increase morbidity and mortality. In addition, obesity can lead to psychological problems such as low self-esteem, anxiety and depression, and physical problems such as shortness of breath, joint pain, oedema and muscle aches. In recent years, studies have shown that being overweight or mildly obese may be associated with a lower risk of death in people aged 80 years and older, a phenomenon known as the “obesity paradox” (25).

To our knowledge, one study (20) has examined the association between weight-adjusted waist circumference index and depression in US adults, but this association has not been examined in older hypertensive patients. In this cross-sectional study, we aimed to use data from the National Health and Nutrition Examination Survey (NHANES) to examine the association between weight-adjusted waist circumference index and depression in older hypertensive patients.

## 2 Materials and methods

### 2.1 Research population

NHANES is a nationally representative survey conducted by the National Center for Health Statistics (NCHS) that uses stratified, multistage probability block sampling to assess the health or nutritional status of the non-institutionalised US population. In this cross-sectional study, we collected publicly available data on participants aged 60 years and older with hypertension from the 2007–2008, 2009–2010, 2011–2012, 2013–2014, and 2015–2016 NHANES cycles with complete and reliable information (demographics, dietary and health-related behaviours, physical measurements, and disease information). The NCHS Research Ethics Review Board approved the NHANES study protocol, and participants provided written informed consent at the time of enrolment. The institutional review board at JNUH determined that the study was exempt because it used publicly identifiable data and waived informed consent. This study followed the Enhanced Reporting of Observational Studies in Epidemiology (Stroboscopic) reporting guidelines.

**Figure 1:**
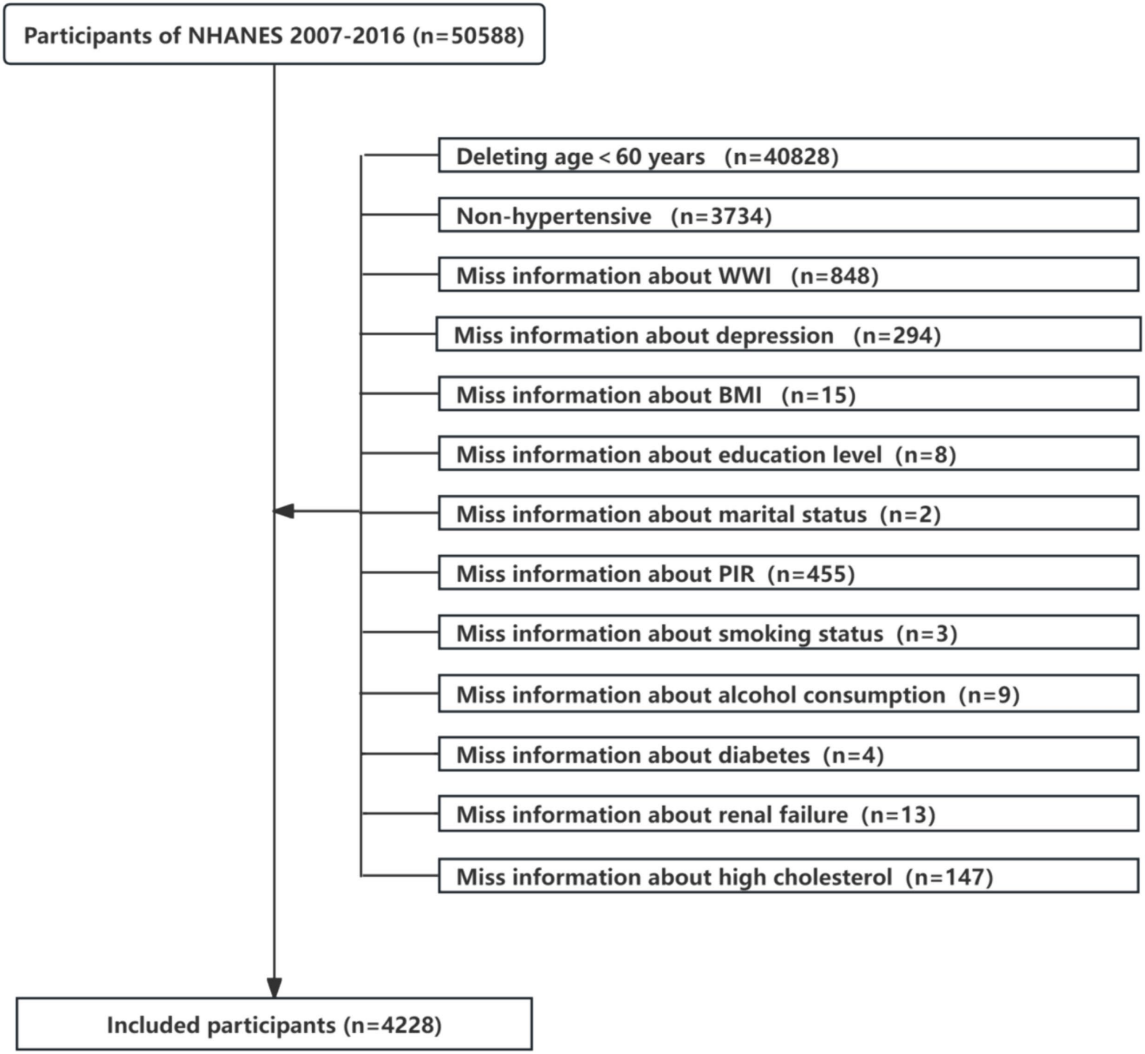
Flowchart for the selection of eligible participants.

This study analysed data from the NHANES 2007–2016 cycle, including the original 50,588 participants. Exclusion criteria for this study included (a) participants aged <60 years (*n* = 40,828), (b) non-hypertensive (*n* = 3,734), (c) participants with incomplete information on WWI (*n* = 848) or depression (*n* = 294), (d) participants with missing data on BMI, education level, marital status, PIR, smoking status, alcohol consumption, diabetes, renal failure, high cholesterol (*n* = 656). A total of 4,228 individuals qualified for analysis, as shown in Figure 1.

### 2.2 Assessment of WWI and depressive symptoms

The WWI index was designed as an exposure variable. WWI=Waist circumference (cm)/Square Root Weight (kg) (26). Depressive symptoms were assessed in this study using the Patient Health Questionnaire-9 (PHQ-9). Trained interviewers administered these questions in the Mobile Examination Centre (MEC) using the Computer Assisted Personal Interview (CAPI) system, which has built-in consistency checks to minimise data entry errors. To ensure the quality of the data, approximately 5% of the interviews were recorded and reviewed for quality control purposes. The PHQ-9 consisted of nine questions related to depressive mood, and the total score was calculated by summing the scores for each item. The response categories for the nine-item instrument, “not at all,” “several days,” “more than half the days” and “almost every day,” were given a score ranging from 0 to 3. The PHQ-9 scale had a range of 0 to 27. A score of 10 or more was considered indicative of a depressive state (27).

### 2.3 Other covariate

A number of potential covariates were assessed according to the literature, including age, body mass index (BMI), sex, race/ethnicity, educational level, marital status, poverty income ratio (PIR), smoking status, alcohol consumption, diabetes, renal failure, high cholesterol and depression. BMI was calculated as weight (kg) divided by height squared (kg/m^2^). Sex (male, female), race (Mexican American, other Hispanic, non-Hispanic black, other race including multiracial, non-Hispanic white). Educational level (less than 9th grade, 9-11th grade, 12th grade without diploma, high school graduate/GED or equivalent, some college or AA degree, college graduate or higher), marital status (married/cohabiting, widowed, divorced, separated, never married), poverty income ratio (PIR), with values categorised as either <1 or ≥ 1 (28). Smoking status was obtained by answering the question ‘Have you ever smoked at least 100 cigarettes in your lifetime? Smoking status was categorised as yes (≥100 cigarettes in lifetime), no (<100 cigarettes in lifetime) (29). Alcohol consumption was assessed by answering the question ‘Have you had at least 12 drinks/1 yr.? Alcohol use was categorised as yes (≥12 drinks/1 yr), no (<12 drinks/1 yr) (29). Diabetes was obtained by answering “Has your doctor told you have diabetes?” and diabetes was categorised as yes, no and borderline (30). The results are based on whether the doctors said yes or no to kidney failure, high blood pressure and high cholesterol.

### 2.4 Statistical methods

The Kolmogorov–Smirnov test was used to determine whether variables were normally distributed. Normally distributed variables were presented as mean (standard deviation), while skewed variables were presented as median (interquartile range, 25–75%). Categorical variables were expressed as proportions (%). Continuous data were compared using t-tests, and categorical data were compared using the χ^2^ test.

WWI was converted from a continuous variable to a binary variable on a 1:1 basis. in the multiple regression model. Model 1 did not adjust for covariates. Model 2 was adjusted for sex, age, race, educational level and marital status, PIR and BMI. Model 3 was adjusted for sex, age, race, education level and marital status, PIR, BMI, smoking and alcohol consumption, and model 4 was adjusted for all variables. We also allowed for smooth curve fitting to account for the non-linearity of the WWI index with depression in older hypertensive patients. When non-linear correlations were observed, a two-band linear regression model (segmented regression model) was used to fit each interval and calculate threshold effects. Subgroup analyses by sex, age and BMI were performed and stratified multiple regression analyses were used. In addition, interaction terms were added to test for heterogeneity of associations between subgroups.

As the sample size was based entirely on the available data, no *a priori* calculation of statistical power was performed. R software (version 4.2.1; R Foundation for Statistical Computing; http://www.Rproject.org), the R survey package (version 4.1-1), and Free Statistics software (version 1.7.1; Beijing Free Clinical Medical Technology Co., Ltd) were used for analyses. In all analyses, a two-tailed *p-*value <0.05 was used to indicate statistical significance.

## 3 Results

### 3.1 Baseline characteristics of the participants

The baseline demographic characteristics of the included participants are shown in Table 1. A total of 4,228 participants were included in this study, of whom 2003 (47.4%) were male and 2,225 (52.6%) were female, with a mean age of 70.2 ± 6.8 years. Characteristics were categorised according to the presence or absence of depression. Age, WWI, waist circumference, BMI, gender, race, education, marital status, PIR, diabetes mellitus, and prevalence of renal failure were significantly different between the two groups (*p* < 0.001), in addition to higher WWI in patients with depression.

**Table 1:** Baselines characteristics of participants.

Characteristic	Total (*n* = 4,228)	Depression (Col%)		*p*-value
		No (*n* = 3,864)	Yes (*n* = 364)	
*Age, year, Mean ± SD*	70.2 ± 6.8	70.4 ± 6.8	67.9 ± 6.3	**< 0.001**
*WWI, Mean ± SD*	11.6 ± 0.7	11.6 ± 0.7	11.8 ± 0.7	**< 0.001**
*Weight,kg, Mean ± SD*	82.5 ± 20.0	82.3 ± 20.0	84.6 ± 20.6	**0.032**
*WC, cm, Mean ± SD*	104.7 ± 14.9	104.5 ± 14.8	107.8 ± 15.6	**< 0.001**
*BMI, kg/m^2^, Mean ± SD*	30.1 ± 6.4	30.0 ± 6.3	31.8 ± 7.1	**< 0.001**
*Gender (n, %)*				**< 0.001**
Male	2003 (47.4)	1875 (48.5)	128 (35.2)	
Female	2,225 (52.6)	1989 (51.5)	236 (64.8)	
*Race (n, %)*				**< 0.001**
Mexican American	469 (11.1)	406 (10.5)	63 (17.3)	
Other Hispanic	403 (9.5)	353 (9.1)	50 (13.7)	
Non-Hispanic white	2074 (49.1)	1923 (49.8)	151 (41.5)	
Non-Hispanic black	1,008 (23.8)	925 (23.9)	83 (22.8)	
Other race	274 (6.5)	257 (6.7)	17 (4.7)	
*Education level (n, %)*				**< 0.001**
<9th grade	623 (14.7)	535 (13.8)	88 (24.2)	
9-11th grade	626 (14.8)	545 (14.1)	81 (22.3)	
High school graduate	1,058 (25.0)	985 (25.5)	73 (20.1)	
Some college or AA degree	1,134 (26.8)	1,038 (26.9)	96 (26.4)	
College graduate or above	787 (18.6)	761 (19.7)	26 (7.1)	
*Marital status (n, %)*				**< 0.001**
Married	2,353 (55.7)	2,222 (57.5)	131 (36)	
Widowed	909 (21.5)	817 (21.1)	92 (25.3)	
Divorced	554 (13.1)	468 (12.1)	86 (23.6)	
Separated	108 (2.6)	87 (2.3)	21 (5.8)	
Never married	209 (4.9)	181 (4.7)	28 (7.7)	
Living with partner	95 (2.2)	89 (2.3)	6 (1.6)	
*PIR (n, %)*				**< 0.001**
<1	769 (18.2)	638 (16.5)	131 (36)	
≥1	3,459 (81.8)	3,226 (83.5)	233 (64)	
*Smoke status (n, %)*				**0.012**
Yes	2,186 (51.7)	1975 (51.1)	211 (58)	
No	2042 (48.3)	1889 (48.9)	153 (42)	
*Consume alcohol (n, %)*				0.953
Yes	2,724 (64.4)	2,490 (64.4)	234 (64.3)	
No	1,504 (35.6)	1,374 (35.6)	130 (35.7)	
*Diabetes (n, %)*				**< 0.001**
Yes	1,230 (29.1)	1,072 (27.7)	158 (43.4)	
No	2,813 (66.5)	2,622 (67.9)	191 (52.5)	
Borderline	185 (4.4)	170 (4.4)	15 (4.1)	
*Failing kidneys (n, %)*				**< 0.001**
Yes	301 (7.1)	253 (6.5)	48 (13.2)	
No	3,927 (92.9)	3,611 (93.5)	316 (86.8)	
*High cholesterol (n, %)*				**0.005**
Yes	2,713 (64.2)	2,455 (63.5)	258 (70.9)	
No	1,515 (35.8)	1,409 (36.5)	106 (29.1)	

WWI, weight-adjusted waist index; BMI, body mass index; WC, waist circumference; PIR, ratio of family income to poverty.

Weighted mean ± standard error for continuous variables: comparisons were performed by the weighted linear regression model. Weighted percentage for categorical variable: comparisons were performed by the weighted chi-square test.

Bold values indicate *p* < 0.05 and results are statistically significant.

### 3.2 A higher WWI index was associated with a higher risk of depression

**Figure 2:**
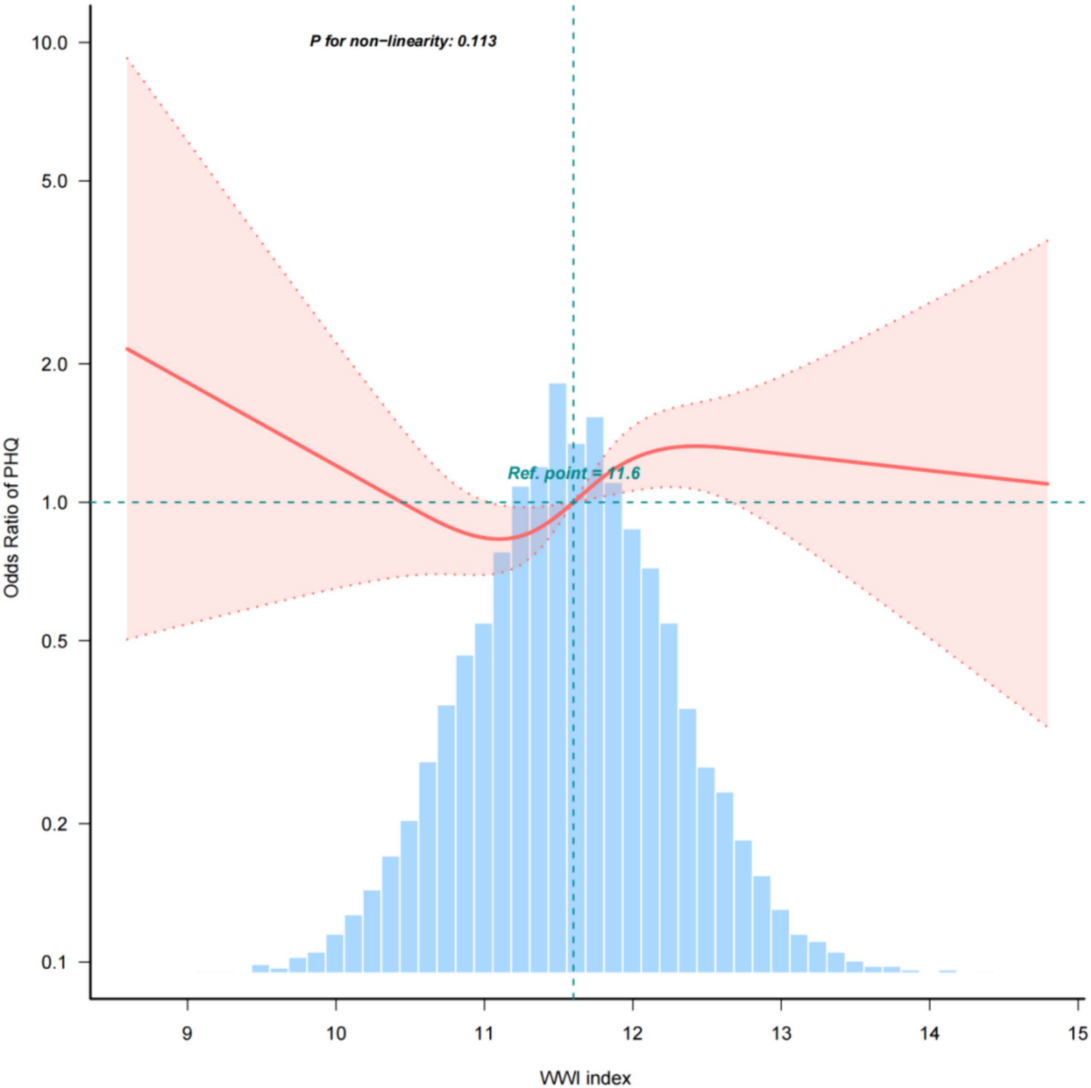
Density, dose–response relationship between the WWI index and the prevalence of depression in older adult hypertensive patients. The area between the upper and lower dashed lines is indicated as the 95% CI. Each point shows the magnitude of the WWI index and is joined to form a continuous line. Adjustments were made for all covariates except effect modifiers (model 4).

Multiple regression analyses with various adjustments for the effect of confounders on the correlation showed that the WWI index was positively associated with the prevalence of depression in the unadjusted model. In the fully adjusted model, each unit increase in the WWI index was associated with a 19% increase in the risk of depression prevalence (OR = 1.19, 95% CI: 0.99, 1.43) (Table 2) The relationship between the WWI index and the prevalence of depression in older adult hypertensive patients was further investigated by smoothed curve fitting. The results showed a non-linear positive correlation between the WWI index and the prevalence of depression in older adult hypertensive patients when the WWI index was ≥11.6 (Figure 2).

**Table 2:** Logistic regression analysis between WWI index with depression in older adult hypertensive patients.

Characteristic	Model 1 OR (95%CI)	Model 2 OR (95%CI)	Model 3 OR (95%CI)	Model 4 OR (95%CI)
WWI	1.51 (1.3 ~ 1.77)	1.28 (1.07 ~ 1.53)	1.27 (1.06 ~ 1.52)	1.19 (0.99 ~ 1.43)
Categories				
Lower (<11.6)	1(Ref)	1(Ref)	1(Ref)	1(Ref)
Higher (≥11.6)	1.9 (1.52 ~ 2.38)	1.52 (1.19 ~ 1.96)	1.51 (1.18 ~ 1.95)	1.43 (1.11 ~ 1.84)
*p*_value	<0.001	0.001	0.001	0.006

WWI, weight-adjusted waist index; BMI, body mass index; WC, waist circumference; PIR, ratio of family income to poverty. OR, odds ratio; CI, confidence interval.

Model 1, no adjusted; Model 2, adjusted for age + gender+BMI + race+education level + marital status + PIR; Model 3, Model 2 + smoke status + consume alcohol; Model 4, Model 3 + diabetes + failing kidneys + high cholesterol.

### 3.3 Subgroup analysis

**Figure 3:**
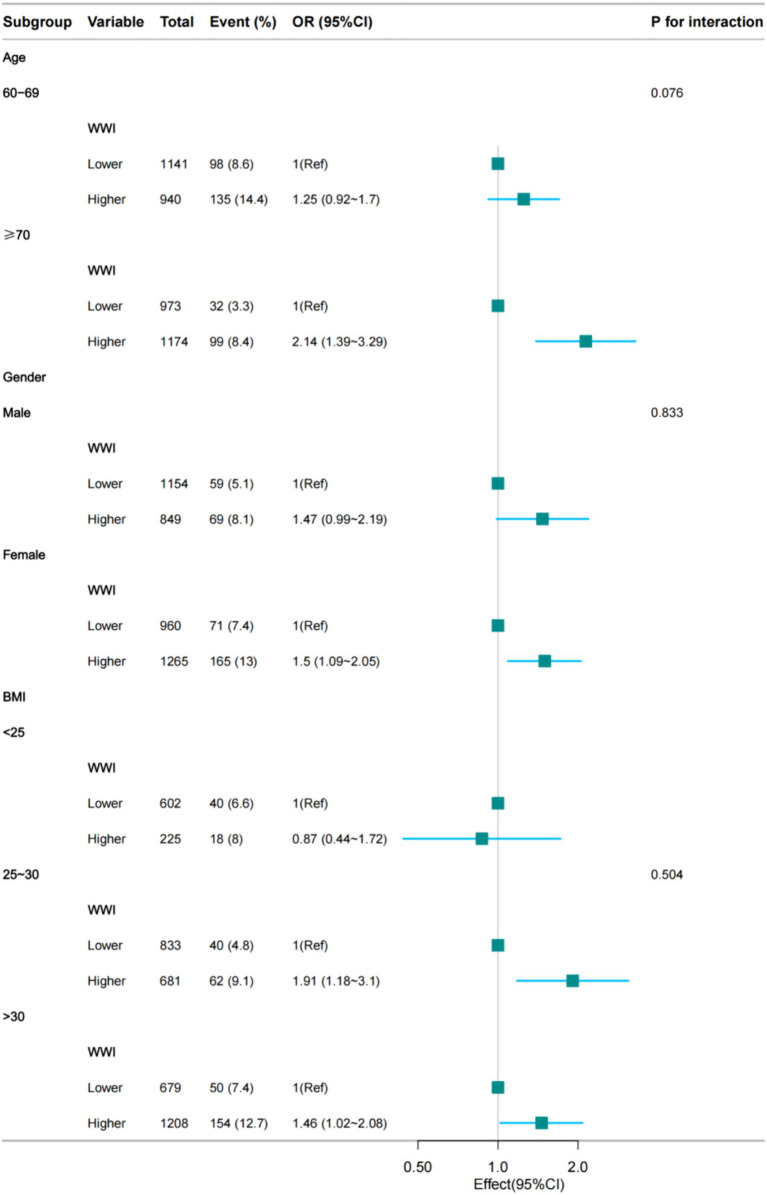
Subgroup regression analysis between the WWI index and the prevalence of hypertensive depression in the older adult (fully adjusted model 4).

We performed subgroup analyses to assess the robustness of the association between the WWI index and the prevalence of depression in older adult hypertensive patients. The results showed significant odds ratios (OR) for demographic groups: 2.14 (95% CI: 1.39, 3.29) for ≥70 years, 1.47 (95% CI: 0.99, 2.19) for men, 1.5 (95% CI: 1.09, 2.05) for women, 1.91 (95% CI: 1.18, 3.1) for BMI 25–30 and 1.46 (95% CI: 1.02, 2.08) for BMI >30. We also tested for interaction with sex, age and BMI. For interaction *p*-values that reached statistical significance, no association was found (Figure 3).

## 4 Discussion

Our study is the first comprehensive analysis of the relationship between the WWI index and the prevalence of depression in older adult hypertensive patients in this large, prospective, nationally representative US population-based study. A 5-cycle population-based study (2007–2016) based on the NHANES database. The results showed that the WWI index was positively associated with the prevalence of depression in older adult hypertensive patients when the WWI index was ≥11.6, with a 19% increased risk of depression prevalence for each unit increase in the WWI index in a fully adjusted model (OR = 1.19, 95% CI: 0.99, 1.43). These results strongly support the value of the WWI index as a predictor of the development of depression in older adult hypertensive patients. We found a 19% higher risk of depression with a high WWI compared with a low WWI. In addition, this association was particularly pronounced in people aged ≥70 years, women and those with a BMI of 25 or more. This is in line with previous findings (20).

Our study found that older adult hypertensive patients aged ≥70 years had a higher risk of depression with increasing levels of the WWI index compared with those aged 60–69 years. With age, a number of changes occur in the biochemical substances involved in mental activities in the brain and in the brain structure of the older adult, leading to a decrease in their ability to withstand setbacks. For example, changes in family roles and status after retirement, loneliness and social isolation, and chronic illness in older people can all trigger depression. In addition, the onset of depression in older people is also associated with major life events, such as the loss of a spouse, relative or friend, retirement, and changes in living environment. These major life variables are no small shock to the mood of older people. At the same time, declining cognitive function, impaired self-esteem and self-confidence are also important factors. Studies have shown (31) that about 50% of older people with depression have comorbid cognitive impairment. Geriatric depression has a variable course and is associated with an increased risk of dementia.

Our study found that older women with hypertension had a higher risk of depression than men as the WWI index increased. Studies have shown that depression is about twice as common in women as in men. This may be related to genetic risk, changes in hormone levels, and other factors (32), and brain structure. For example, women are more likely to experience depression during puberty, pregnancy and the menopause because of changes in hormone levels. In addition, studies have shown that decreasing levels of oestrogen and increasing levels of follicle-stimulating hormone are risk factors for depression in menopausal women. Although men are diagnosed with depression at half the rate of women, they are three to four times more likely to die by suicide than women (33). So for men with depression, we need to be equal.

Our study found that overweight or obese older adult hypertensive patients had a higher risk of depression with increasing levels of the WWI index compared to normal weight. A BMI >25 is considered to be overweight or obese. People who are overweight or obese may suffer from social discrimination, negative stereotyping and weight bias, which can lead to lower self-esteem and social isolation, which in turn increases the risk of depression. Obesity can be associated with unhealthy eating habits, physical inactivity and sleep problems, all of which are risk factors for depression. Obesity increases the risk of a number of chronic diseases, such as diabetes, cardiovascular disease and arthritis, which have been linked to the development of depression.

The specific biological mechanisms linking the Weight Waist Adjustment Index (WWI) and depression are not yet fully understood, but researchers have suggested some possible pathways. Inflammatory response. Excess abdominal fat is associated with chronic low-grade inflammation. Inflammatory factors released from adipose tissue, such as tumour necrosis factor-a (TNF-a) and interleukin-6 (IL-6), can travel through the bloodstream to the brain and cause depression (34). Endocrine changes. Abdominal fat can lead to changes in hormone levels, such as insulin resistance (35) and increased levels of cortisol (a stress hormone). Changes in these hormones can affect mood and behaviour, leading to depression. Neurotransmitter imbalance. Obesity can affect levels of neurotransmitters in the brain, such as serotonin, dopamine and noradrenaline (36). These are important neurotransmitters that regulate emotions and mood and can lead to depression. Altered brain structure. Chronic obesity can lead to changes in brain structure and function, such as a reduction in the volume of the hippocampus and prefrontal cortex, areas closely linked to mood regulation and cognitive function, which can lead to depression. Oxidative stress. Obesity may be associated with increased oxidative stress (37), Oxidative stress is an imbalance between free radical production and antioxidant defences, which can lead to cellular damage and brain dysfunction, resulting in depression. Metabolic disorders. Abdominal obesity is often associated with metabolic syndrome, a condition that involves multiple metabolic abnormalities, including hypertension, hyperglycaemia, hyperglycaemia and other disorders, and these disorders may be associated with the development of depression. Genetics and geneticists. Obesity and depression may share a common genetic basis. In addition, energetic changes such as the RNA (38). May affect gene expression associated with obesity and depression. Gut macrobiotic imbalance. Obesity may affect the composition of the macrobiotic gut, and there is a link between the macrobiotic gut and brain health, known as the ‘gut-brain axis’, which may lead to depression (34). These mechanisms are not independent of each other; they may interact and work together to cause depression. However, more research is needed to clarify the exact roles of these pathways and their interactions.

The Weight Waist Circumference Adjustment Index (WWI) can be used as a tool to assess and prevent the risk of depression. For individuals with a high WWI, the risk of depression should be assessed; a high WWI may be a warning sign that further mental health assessment is needed. A combination of the WWI and other mental health screening tools, such as the Patient Health Questionnaire (PHQ-9), are used to assess the severity of depressive symptoms. People with high WWI scores and depressive symptoms are advised to seek professional medical advice. Long-term follow-up of WWI and depressive symptoms to monitor the effect of the intervention. If the WWI decreases, monitor whether depressive symptoms improve; for high-risk individuals, a combination of interventions, including lifestyle changes, psychotherapy and medication as needed. The WWI is a new type of obesity index calculated by dividing waist circumference (in centimetres) by the square root of weight (in kilograms). In the US population, the 2nd and 3rd quartiles of the WWI are 10.41–11.01 and 11.01–11.60, respectively, when calculated in quartiles. This means that WWI values between 10.41 and 11.60 can be used as a reference range for assessing the risk of obesity and depression (20). Measure your weight and waist circumference regularly and calculate your WWI to monitor your health. If your WWI is outside the normal range, you may be at risk of abdominal obesity and need to take action. Live a healthy lifestyle. Maintain a healthy WWI by eating a balanced diet and exercising regularly, including plenty of vegetables, fruit, whole grains and lean protein, and limiting foods high in sugar and fat. Regular aerobic exercise and resistance training to reduce belly fat. Set realistic weight and waist circumference goals to reduce abdominal fat. Achieve goals gradually and avoid extreme or unhealthy weight loss methods. Understanding the potential link between abdominal obesity and depression and raising awareness of the importance of a healthy lifestyle; however, when using WWI to prevent and assess depression, it is important to remember that WWI is only one of many health indicators and that preventing and treating depression requires a combination of an individual’s overall health, lifestyle, psychosomatic factors and medical history.

Our study has several strengths. First, the participants in NHANES were a representative sample from the United States who followed a carefully designed study protocol with rigorous quality control and assurance to ensure that our conclusions are reliable. Second, NHANES provided extensive demographic and metabolic data and an average follow-up of more than 23 years. We adjusted for important confounders in our multiple regression analyses. In addition, we adjusted for confounding variables and performed subgroup analyses to ensure that our results were applicable to a wider range of individuals. However, there are some limitations to our study. First, our study was a cross-sectional study, and we were not able to clarify the causal relationship between the WWI index and the prevalence of hypertensive depression in older adults. Second, the survey data from NHANES were based on questionnaires, which means that recall bias may exist. Despite these limitations, this paper demonstrates for the first time the relationship between the WWI index and depression in older hypertensive patients and provides strong support for the WWI index as a predictor of the development of depression in older hypertensive patients. Further studies, especially in low-and middle-income countries using longitudinal cohorts, are therefore needed for further validation.

## 5 Conclusion

This study suggests that older hypertensive patients have a WWI index of 11.6 ± 0.7. When the WWI index is ≥11.6, elevated WWI scores are associated with a higher likelihood of depression in older hypertensive patients, especially in women. Assessment by WWI index level may be beneficial in reducing the risk of developing depression, and the benefit may be greater especially for those aged ≥70 years.

## Data Availability

The datasets presented in this study can be found in online repositories. The names of the repository/repositories and accession number(s) can be found in the article/supplementary material.
